# Physicochemical and Antibacterial Properties of Conventional and Two Premixed Root Canal Filling Materials in Primary Teeth

**DOI:** 10.3390/jfb13040177

**Published:** 2022-10-06

**Authors:** Claire El Hachem, Jean Claude Abou Chedid, Walid Nehme, Marc Krikor Kaloustian, Nabil Ghosn, Hafsa Sahnouni, Davide Mancino, Youssef Haikel, Naji Kharouf

**Affiliations:** 1 Department of Pediatric Dentistry, Faculty of Dentistry, Saint Joseph University, Beirut 1107 2180, Lebanon; 2 Department of Endodontics, Arthur A. Dugoni School of Dentistry, University of the Pacific, 155 5th Street, San Francisco, CA 94103, USA; 3 Department of Endodontics, Faculty of Dentistry, Saint Joseph University, Beirut 1107 2180, Lebanon; 4 Craniofacial Research Laboratory, Faculty of Dental Medicine, Saint Joseph University, Beirut 1107 2180, Lebanon; 5 Department of Biomaterials and Bioengineering, INSERM UMR_S 1121, Biomaterials and Bioengineering, 67000 Strasbourg, France; 6 Department of Endodontics, Faculty of Dental Medicine, Strasbourg University, 67000 Strasbourg, France; 7 Pôle de Médecine et Chirurgie Bucco-Dentaire, Hôpital Civil, Hôpitaux Universitaire de Strasbourg, 67000 Strasbourg, France

**Keywords:** deciduous tooth, calcium silicate material, calcium hydroxide and iodoform, zinc oxide eugenol, root canal filling

## Abstract

In this study, some physicochemical and antibacterial properties of three root canal filling materials for primary teeth, Calplus “CP” (Prevest DenPro, Lewes, DE, USA), Bio-C Pulpecto “Bio-CP” (Angelus, Basil, Londrina, Paraná, Brazil), and Zinc Oxide and Eugenol “ZOE” (Prevest DenPro, Lewes, DE, USA) were compared. For each material, the pH, solubility, contact angle, and crystalline microstructure under SEM were evaluated. Their antibacterial activity against *Enterococcus faecalis* was determined through direct tests. The Kruskal–Wallis test was used to analyze the results using a one-way analysis of variance on ranks. All the materials had an alkaline pH at 3, 24, and 72 h, with CalPlus having the highest (*p* < 0.05). Bio-CP was more soluble during the evaluation period (24 h) than ZOE and CalPlus (*p* < 0.05). Bio-CP and ZOE demonstrated the creation of crystallite structures on their surfaces after immersion in PBS at 37 °C, whereas CalPlus showed none. The lowest contact angle was observed for Bio-CP (53 ± 1.5°); contact angles of (86 ± 4°) and (96 ± 1°), respectively, were observed after 10 s of the deposition of the water drop for CalPlus and ZOE. In conclusion, according to this study, there is still a need to develop new filling materials for primary teeth. ZOE, CalPlus and Bio-CP demonstrated different physicochemical and antibacterial properties, but none of the materials had optimal properties and could be considered the most suitable filling material for primary teeth pulpectomy. Bioceramics in their current state are not an alternative. The physicochemical and antibacterial properties still need improvement to fit the intricate anatomy of primary teeth.

## 1. Introduction

When faced with an irreversibly infected or necrotic pulp, pulpectomy aims to retain primary teeth in a functional, healthy state until their physiological exfoliation [1]. The ultimate root canal filling material should be antibacterial, and resorbable at the same rate as the primary roots or if extruded. Moreover, it should bond to canal walls without shrinking, and cause no harm to the periapical area or the developing tooth germ [2].

A plethora of materials have been proposed over the years like zinc oxide eugenol alone or with formocresol, iodoform, and camphorated phenol. Pastes containing calcium hydroxide, iodoform, or a mixture of both have also been suggested [3,4,5,6].

Zinc oxide eugenol (ZOE) cement, a combination of zinc oxide (powder) and eugenol (liquid) has been, until recently, the most common filling material used [7]. However, it sets into a thick mass that resists resorption, may irritate periapical tissues, and can cause deviation of the permanent tooth bud [8].

Oil-based calcium hydroxide pastes containing iodoform, such as CalPlus (DenPro, Prevest, USA), have also been advocated as filling materials for primary teeth due to their antibacterial and healing properties, and easy handling [9]. When compared to the conventional filling materials (ZOE), this product is premixed and pre-dosed. It does not require any manual mixing procedure [10]. Their main disadvantage is potential external resorption and intracanal resorption, which can ultimately lead to failure [11].

New endodontic types of cement, such as bioceramics, have been gaining in popularity because of their physicochemical and biological properties, including alkaline pH, shrink-free property, chemical stability in the biological environment, biocompatibility, and bioactivity [12]. Bioceramics are composed mainly of calcium silicate materials and are a substitute for the traditionally used calcium hydroxide [13]. These bioactive materials are used in permanent teeth in different applications such as pulpotomy, pulp capping, resorption, perforation repair, and root canal filling [14,15,16,17]. They have also been recently advocated in pediatric dentistry [18].

Bio-C Pulpecto (Bio-CP) (Angelus, Basil, Londrina, Paraná, Brazil) is the first resorbable bioceramic root canal filling material for primary teeth. It is composed of titanium oxide, ester glycol salicylate, silicon dioxide, calcium tungstate, toluene sulphonamide, and calcium silicate [19]. To date, there is no comparative study between Bio-CP, ZOE and CalPlus paste regarding their physicochemical and antibacterial properties.

The obturation technique of primary teeth relies solely on the filling material, hence the importance of the antibacterial activity of the filling material. One of the most prevalent species resistant to mechanical preparation and irrigation protocols identified in human primary teeth is *Enterococcus faecalis* [20,21,22]. Moreover, when opting for a filling material, there should be a complete understanding of the physical and chemical behavior of the material in order to choose the most appropriate for every clinical situation.

The aim of this study was to evaluate, in vitro, some physicochemical properties and the antibacterial activity of ZOE, CalPlus, and Bio-C Pulpecto as resorbable filling materials to determine the most appropriate filling material for primary teeth pulpectomy. The null hypothesis was that there would be no significant difference in the physicochemical and/or antibacterial characteristics between the three tested materials.

## 2. Materials and Methods

### 2.1. Materials

Calplus “CP” (DenPro, Prevest, USA), Bio-C Pulpecto “Bio-CP” (Angelus, Basil, Londrina, Paraná, Brazil), and Zinc Oxide and Eugenol “ZOE” (DenPro, Prevest, USA) were used in the present study following the manufacturer’s instructions (Table 1). All specimens were conserved in a dark container for 48 h at 37 °C to accomplish a complete setting time [23].

### 2.2. pH Measurements of the Aqueous Solution in Contact with the Cement

For each group, five samples were prepared using Teflon molds (3 mm in diameter and 3.8 in height). Each sample was put in contact with 10 mL of distilled water at 37 °C. A pH meter “CyberScan pH 510” (Thermo Scientific, Waltham, MA, USA) was used to measure the pH of water in contact with each sample at 3, 24, and 72 h. Distilled water was flushed over the pH meter electrode to eliminate contamination from the previous solution.

### 2.3. Solubility

Three samples (20 mm in diameter and 2 mm in height) of each group were prepared and analyzed following a previous study [24]. Using a digital system (accuracy ± 0.0001 g), the samples were weighed three times before the 24 h of the immersion period in 50 mL of distilled water at 37 °C. After the aging period, the samples were taken out from distilled water and then dried at 37 °C for 24 h. Finally, the samples were weighed again three times and averaged to obtain the final weight. For each material, the solubility percentage was attained from the difference in mass between the final and the initial weight.

### 2.4. Scanning Electron Microscope (SEM) of Crystallites Creation

Twelve samples for each material were prepared as described in Section 2.2. From each group, three samples were stored in hermetic boxes and kept in dry conditions. The remaining samples from each group were put in 10 mL of phosphate-buffered saline (PBS10×, Dominique Dutscher, Bernolsheim, France) at 37 °C. Three periods were assigned (24, 72, and 168 h) to investigate the morphological changes of the cement surfaces using SEM analysis. After each period, distilled water was poured over the samples for 5 min. These were mounted on SEM stubs and sputter-coated with gold–palladium (20/80), then analyzed using an SEM (FEI Company, Eindhoven, The Netherlands, 10 kV) at a magnification of ×5000 with a working distance of 10 mm [25].

### 2.5. Water Sorption Tests

From each group, three samples were prepared using Teflon molds (10 mm in diameter and 2 mm in height). After the setting time, the samples were kept dry in the fume hood overnight. To evaluate the sorption time of a 5 µL drop of distilled water into the cement surface, a contact angle device (Biolin Scientific, Espoo, Finland) was used [14]. Using a horizontal camera, a movie was recorded to track the profile of the water drop and its absorption time.

### 2.6. Antimicrobial Activity

*Enterococcus faecalis* (*E. faecalis*, ATCC 29212) was cultured in a Brain Heart Infusion medium (BHI) (Darmstadt, Germany). The turbidity of the bacterial medium containing *E. faecalis* was adapted at OD_600(nm)_ = 0.3. To evaluate the antibacterial activity of these against *E. faecalis*, a direct contact test (DCT) was used. Each sample was placed in a well (24-well culture plate) (in triplicate). One mL of the bacterial medium was added to each well and incubated in anaerobic conditions at 37 °C for 24 h under constant stirring at 450 rpm [26]. The bacterial medium without the tested filling materials was used as a control group. After 24 h, on each specimen from each group, 10-fold serial dilutions up to 10^6^ in BHI were performed. A volume of 100 µL of each diluted medium was added onto a BHI agar plate, then homogeneously spread and incubated at 37 °C for 24 h. Manual counting was used to measure the *E. faecalis* concentration by counting the colonies on the plate, and their CFU/mL (colony forming units/mL) was determined.

### 2.7. Statistical Analysis

The SigmaPlot release 11.2 (Systat Software, Inc., San Jose, CA, USA) was used to analyze the data. The normality of the data was verified with the Shapiro–Wilk test. To determine whether significant differences existed in the antibacterial activity, pH values, solubility, and angle contact, the Kruskal–Wallis test (one-way analysis of variance on ranks) including multiple comparison procedures (Tukey Test) was used. A statistical significance level was set at α = 0.05.

## 3. Results

### 3.1. pH Analysis

**Figure 1:**
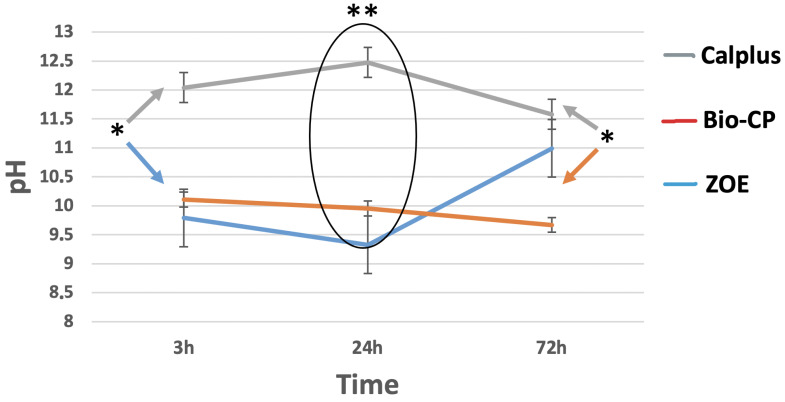
pH changes of the water in contact with the different tested types of cement after 3, 24, and 72 of immersion in distilled water at 37 °C. Calplus (CP), Bio-C Pulpecto (Bio-CP), and Zinc Oxide and Eugenol (ZOE). (* *p* = 0.002; ** *p* ≤ 0.001).

The pH of the solution in contact with the different types of cement was described for 72 h in Figure 1. All three types of cement demonstrated an alkaline pH for the solution in contact at 3, 24, and 72 h. CalPlus demonstrated the highest alkaline pH during 72 h compared to the two other types of cement. At 3 h, CalPlus has higher alkaline pH than ZOE (*p* < 0.05). At 24 h, a significant difference was found between the three tested materials (*p* < 0.05). Finally, at 72 h, ZOE has a higher pH than Bio-CP with no difference compared to CalPlus.

### 3.2. Solubility

**Figure 2:**
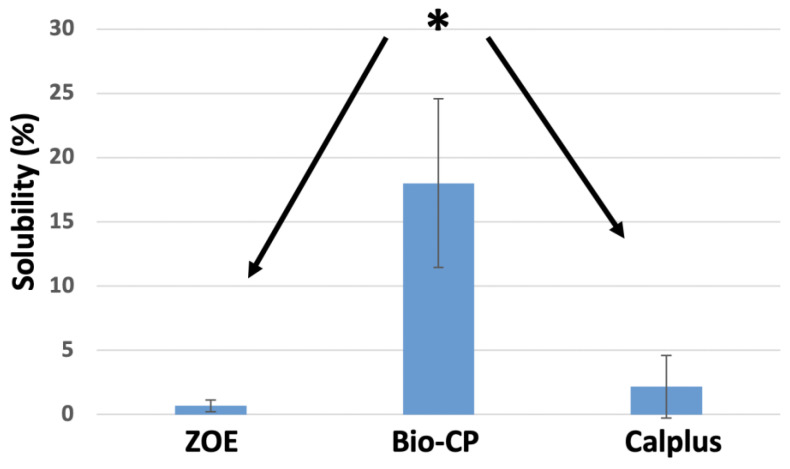
Solubility percentages of the different types of cement after immersion in 50 mL of distilled water at 37 °C for 24 h. * *p* < 0.05.

For each tested material, the mean and standard deviation of solubility (wt.%) values are presented in Figure 2. Bio-CP was more soluble during the evaluation period (24 h) than ZOE and CalPlus (*p* < 0.05).

### 3.3. Scanning Electron Microscope (SEM)

**Figure 3:**
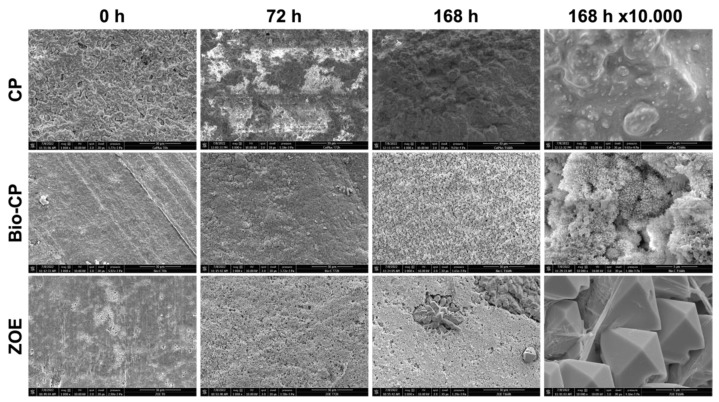
Scanning electron microscope images (×1000 and ×10,000 magnifications) demonstrate the morphological changes of each material at different time points of immersion in phosphate-buffered solution at 37 °C.

The crystalline structures of the three types of cement are shown in Figure 3. Bio-CP and ZOE demonstrated the creation of crystallite structures on their surfaces after immersion in PBS at 37 °C. Some zones of ZOE surface demonstrated cubical crystalline structures, whilst Bio-CP showed an urchin-like crystallite structure. CalPlus did not show any crystallite creation.

### 3.4. Water Sorption Tests

**Figure 4:**
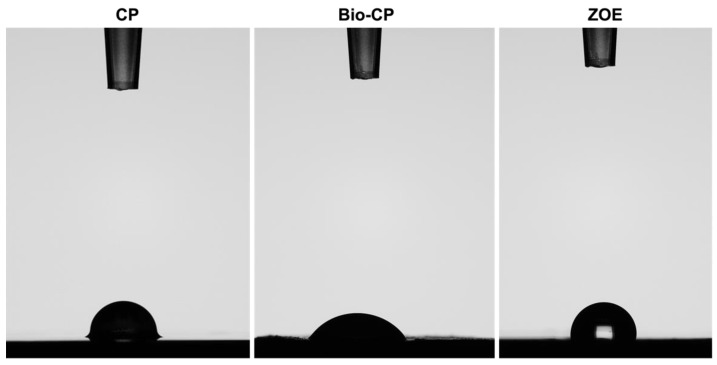
Contact angles after 5 μL of water dropped on the different cement surfaces after 10 s of deposition. Calplus (CP), Bio-C Pulpecto (Bio-CP), and Zinc Oxide and Eugenol (ZOE).

Bio-CP demonstrated the highest hydrophilicity for 5 uL of a drop of distilled water compared to ZOE and CalPlus. The lowest contact angle was observed for Bio-CP (53 ± 1.5°). Contact angles of (86 ± 4°) and (96 ± 1°), respectively were observed after 10 s of deposition of the water drop for CalPlus and ZOE (Figure 4, Table 2).

### 3.5. Antimicrobial Activity

**Figure 5:**
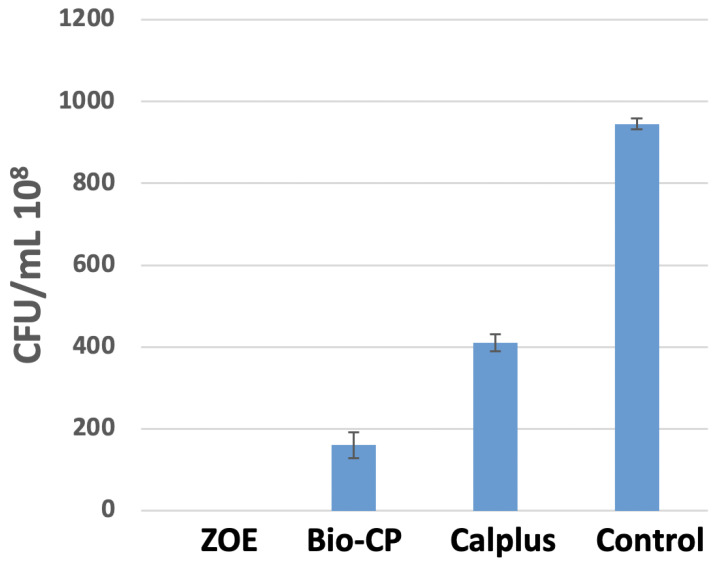
The number of colonies forming units/mL of *Enterococcus faecalis* for the medium in contact with Calplus “CP”, Bio-C Pulpecto “Bio-CP”, Zinc Oxide, and Eugenol “ZOE” and the control group “C” (bacterial medium) after 24 h at 37 °C in anaerobic conditions.

Significant bacterial growth was observed after 24 h between all the groups (*p* < 0.05). ZOE is the most effective, killing 100% of bacteria, followed by Bio-CP, which eliminates more than 75%, and finally CalPlus (more than 50% of bacteria) (*p* < 0.05) (Figure 5). In addition, all three types of cement have antibacterial activity against *E. faecalis* and the best-performing cement was ZOE.

## 4. Discussion

The obturation of primary teeth relies completely on the filling material [27]. ZOE has been, until 2008, the only material explicitly recommended by the American Academy of Pediatric Dentistry (AAPD) [28]. However, it cannot be regarded as an ideal root canal filling due to its resorption rate and negative effects when extruded [3]. Calcium hydroxide and iodoform pastes were recently recommended and showed a high success rate in randomized clinical trials [29]. Nonetheless, in a recent systematic review and meta-analysis, an unclear or high risk of bias was proven in most of the studies and the overall certainty of the evidence ranged from low to very low, denoting that there is still no conclusion to be drawn as to the best pulpectomy material [9].

A resorbable Bioceramic paste, like Bio-CP, could be a promising alternative if the material possessed physicochemical and antibacterial properties suitable for the filling of the intricate root canal system of primary teeth [30]. This was the first in vitro study in the pediatric endodontics literature to focus on the pH, solubility, contact angle, crystallographic changes using SEM images after immersion in PBS, and antibacterial activity of three filling materials for primary teeth. The results of this study showed significant differences between ZOE, CalPlus, and Bio-CP for all evaluated criteria; thus, the null hypothesis was rejected.

The pH analysis indicated an alkaline pH for all tested materials in contact with distilled water at 24 h. An alkaline pH plays a role in the deposition of mineralized tissue and neutralizing the lactic acid from the osteoclast, and promoting healing [31]. Higher pH values were observed for CalPlus compared to ZOE and Bio-CP (*p* < 0.05), which can be attributed to its dissociation into Ca^2^+ and OH^−^ ions and has already been validated in many previous studies [32,33]. However, the main disadvantage of this type of material is intracanal resorption and accelerated external root resorption reported in several clinical studies [27].

A set endodontic sealer should present solubility of less than 3% according to ISO standards [23]. In our study, Bio-CP exhibited very high solubility exceeding 3%, demonstrating a high level of Ca^2+^ and bioactivity. However, high solubility also means that the material dissolves quickly, creating gaps in the canals and generating reinfection [34]. This was also observed in other studies about bioceramic sealers [35] and could be explained by the presence of fine hydrophilic particles in the composition of these sealers, which generate an increase in surface area that may increase sealer solubility when in contact with water/moisture, [36]. Therefore, it is essential to determine clinically the rate at which Bio-CP dissolves, since there is no Gutta Percha to complement the sealer in the root canal treatment of primary teeth. Moreover, the lifespan of a root canal treatment varies greatly depending on the child’s age and physiologic stage of the roots. In the only other study about Bio-CP, the authors highlighted that Bio-CP exhibited sufficient physicochemical properties, disclosed cytocompatibility, and indicated the potential to stimulate mineralization, but lacked clinical support [19].

Using SEM analysis, the microstructural crystalline formation during the initial setting time in 95% humidity and after 3 and 7 days in water was observed. CalPlus did not demonstrate a clear crystallite formation on its surface (Figure 3). This could be because this material is a non-setting paste and the acquisition of such an image was not possible. This result could not be compared to other studies since, to the best of our knowledge, there has not been a publication about the microstructure of calcium hydroxide iodoform-based materials.

The formation of a crystalline structure stipulates that remineralization can occur [23]. In contrast, ZOE had some zones with cubical structures, whereas Bio-CP had an urchin-like structure on its surface. This aspect was extensively described in many studies and was attributed to the extraction of hydroxyapatite from natural resources [37,38].

Contact angle measurements are a reliable tool to better understand the interactions between solids and liquids [39]. These interactions play a major role in explaining not only material wettability, but also wetting, spreading, and adsorption of liquids [39]. In this study, Bio-CP had the lowest contact angle (53 ± 1.5°), demonstrating a good wetting ability, and the capacity to spread faster on substrates such as dentin [40]. CalPlus and ZOE both exhibited poor surface wetting with high contact angles of (86 ± 4°) and (96 ± 1°), respectively. Tummala et al. also found a high contact angle for ZOE and credited this to the increased viscosity of the sealer [41].

The antibacterial potential of each material against *E. faecalis* was evaluated using a direct contact test per previous studies [42]. Anaerobic bacteria were found scattered within the whole root canal system of primary teeth (accessory canals, dentinal tubules, secondary canals, apical foramen) as well as on the physiological resorptive zones, hence the crucial role of the antibacterial property of the filling paste [43]. This species of bacteria may persist even after biomechanical preparation and use of intracanal irrigants [44]. According to this study, ZOE, CalPlus, and Bio-CP possess antibacterial activity compared to the bacterial medium (*p* < 0.05). ZOE is the most efficient (*p* < 0.05), corroborating the results of previous studies [45]. Eugenol-based root canal filling materials possess antibacterial activity due to the action of eugenol, triggering protein denaturation and rendering the microorganisms non-functional [46]. However, in some studies, ZOE demonstrated the smallest zones of bacterial growth inhibition against *E. faecalis* [43], and higher antibacterial activity for iodoform pastes was observed [47].

CalPlus iodoform paste did not set; therefore, compressive tests were not carried out. Furthermore, the filling ability, flow, radio-opacity and biological properties of each material were omitted [23], and they are important factors to consider. Further in-vitro studies should be conducted to better understand the physical and chemical properties of filling materials for primary teeth. Ageing of Bio-CP in simulated body fluid could provide interesting data about the resorption rate compared to that of the roots of primary teeth [48].

The results obtained in this study are encouraging for the use of resorbable bioceramics in pediatric endodontics. Bio-CP contains calcium silicate and can be classified as a bioactive material. It is capable of forming hydroxyapatite or carbonated apatite on its surface and inducing osteogenesis [48].

However, the results of this study are not conclusive and there is still a need to conduct more well-designed studies to better understand the credentials of an ideal filling material. For the Bioceramics, there is still a need to improve their properties, mainly the excessive solubility, and to increase their antibacterial effect. Moreover, due to the continuous root resorption occurring on primary teeth, there is a need for randomized long-term clinical trials to assess the clinical behavior of this type of material.

## 5. Conclusions

Within the limitations of this in-vitro study, it could be concluded that there is still a need to develop new filling materials for the root canal treatment of primary teeth. Given the results of this study, ZOE, CalPlus and Bio-CP demonstrated different physicochemical and antibacterial properties, but none of the materials had optimal properties and could be considered the most suitable filling material for primary teeth pulpectomy. The properties of bioceramics such as bioactivity, solubility in fluids, and adhesiveness would provide a crucial step in increasing the success rate of root canal treatment on primary teeth and developing more performant materials. More research should be conducted to optimize clinical protocols in pediatric endodontics.

## Figures and Tables

**Table 1:** Manipulation and manufacturer and of tested materials.

Materials	Manufacturer	Lot	Mixing
Calplus (CP)	Prevest DenPro, Lewes, DE, USA	3121906	Premixed
Bio-C Pulpecto (Bio-CP)	Angelus, Basil, Londrina, Paraná, Brazil	51152	Premixed
Zinc Oxide and Eugenol (ZOE)	Prevest DenPro, Lewes, DE, USA	1561912	1.4 g zinc oxide to 0.4 mL eugenol

**Table 2:** Contact angles of 5 µL of distilled water on the different material surfaces after 10 s of deposition. Statistical significance (*p* < 0.05) is indicated with superscript letters a, b and c. Calplus (CP), Bio-C Pulpecto (Bio-CP), and Zinc Oxide and Eugenol (ZOE).

Test\Materials	CalPlus	Bio-CP	ZOE	Statistical Significance
**Contact angle (°)**	86 ± 4 ^a^	53 ± 1.5 ^b^	96 ± 1 ^c^	*p* < 0.05

## Data Availability

Not applicable.
